# Unwrapping NPT Simulations to Calculate Diffusion Coefficients

**DOI:** 10.1021/acs.jctc.3c00308

**Published:** 2023-05-31

**Authors:** Jakob Tómas Bullerjahn, Sören von Bülow, Maziar Heidari, Jérôme Hénin, Gerhard Hummer

**Affiliations:** † Department of Theoretical Biophysics, Max Planck Institute of Biophysics, 60438 Frankfurt am Main, Germany; ‡ Structural Biology and NMR Laboratory, Linderstrøm-Lang Centre for Protein Science, Department of Biology, University of Copenhagen, 2200 Copenhagen, Denmark; ¶ Laboratoire de Biochimie Théorique UPR 9080, Institut de Biologie Physico-Chimique, CNRS and Université Paris-Cité, 75005 Paris, France; § Institute of Biophysics, Goethe University Frankfurt, 60438 Frankfurt am Main, Germany

## Abstract

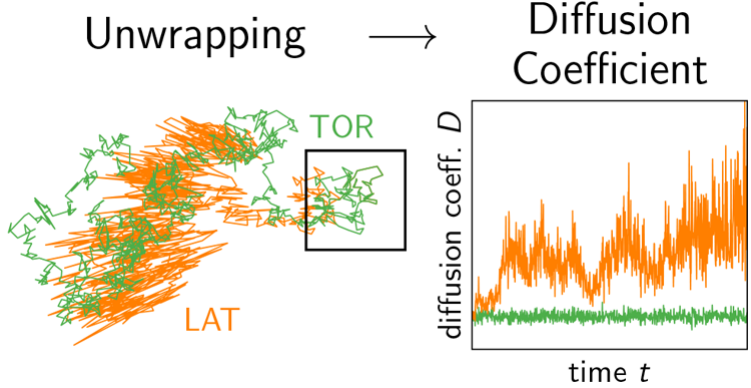

In molecular dynamics simulations in the NPT ensemble at constant pressure, the size and shape of the periodic simulation box fluctuate with time. For particle images far from the origin, the rescaling of the box by the barostat results in unbounded position displacements. Special care is thus required when a particle trajectory is unwrapped from a projection into the central box under periodic boundary conditions to a trajectory in full three-dimensional space, e.g., for the calculation of translational diffusion coefficients. Here, we review and compare different schemes in use for trajectory unwrapping. We also specify the corresponding rewrapping schemes to put an unwrapped trajectory back into the central box. On this basis, we then identify a scheme for the calculation of diffusion coefficients from NPT simulations, which is a primary application of trajectory unwrapping. In this scheme, the wrapped and unwrapped trajectory are mutually consistent and their statistical properties are preserved. We conclude with advice on best practice for the consistent unwrapping of constant-pressure simulation trajectories and the calculation of accurate translational diffusion coefficients.

## 1 Introduction

Molecular dynamics (MD) simulations are performed by numerically solving the classical equations of motion for every particle in a given system. For systems in the condensed phase, such as proteins in water, these simulations are usually conducted in volumes of finite size subject to periodic boundary conditions (PBCs). In constant-volume simulations, one can think of the periodic system either as a single box in which opposite faces are identified under what are also referred to as toroidal boundary conditions, or as an infinite periodic lattice of replicates of the central simulation box. In the *toroidal view*, a particle leaving the central simulation box placed at the coordinate origin re-enters the box at the opposing face, as it would when moving around on a torus. In the *lattice view*, each particle corresponds to a collection of infinitely many points on a periodic lattice, whose lattice constants are determined by the box size and shape. The toroidal view naturally leads to so-called wrapped trajectories, where particles at every instance in time are contained within the central box (and positions outside the box do not make mathematical sense). By contrast, in the lattice view each individual marked point on the lattice representing a particular particle can traverse the full three-dimensional space, resulting in an associated unwrapped trajectory. For simulation boxes of constant volume in constant-energy (NVE) and constant-temperature (NVT) ensembles, the task of unwrapping a trajectory therefore corresponds to transforming from the toroidal view to the lattice view.

**Figure 1:**
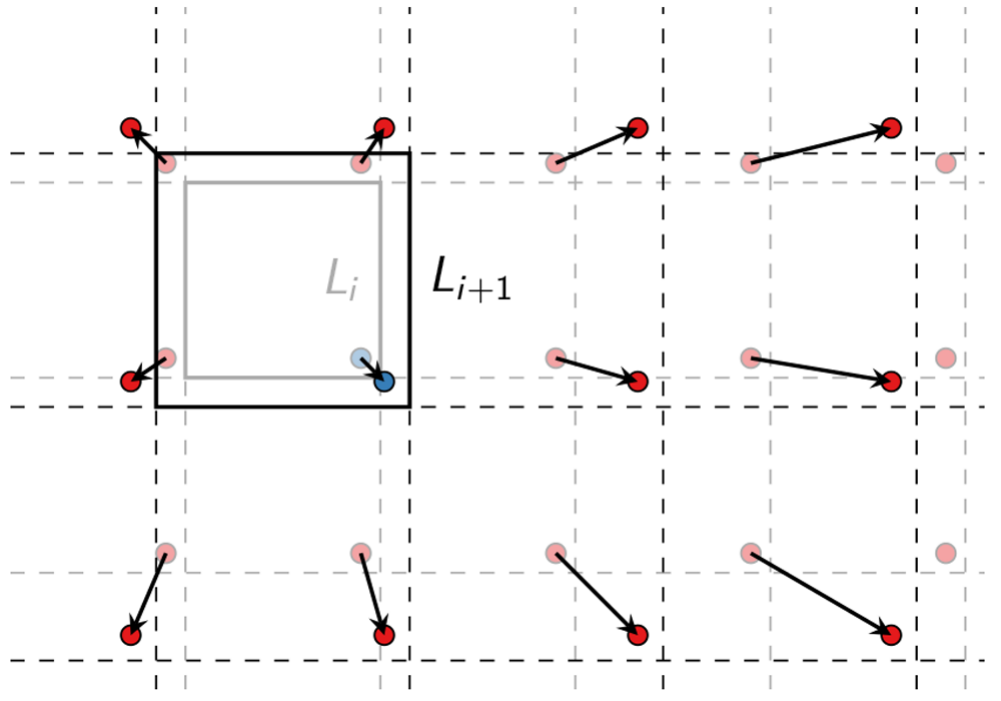
Barostat box rescaling in lattice view of PBCs. In the lattice view, the displacement resulting from barostat-induced rescaling of the box volume grows with the distance from the reference box centered at the coordinate origin. The central boxes before and after barostat action are indicated by gray and black squares, respectively, and the corresponding periodic images of a particle by circles with faint and solid colors. As a result of barostat rescaling alone, particle images (red) away from the central box move farther than the reference particle (blue), as indicated by the arrows.

In constant-pressure (NPT) simulations, however, the task of unwrapping becomes somewhat ambiguous, because the barostat constantly modifies the size and shape of the simulation box to keep the average pressure fixed. The positions of the particles within the box thereby get rescaled.^1^ In the lattice view of PBCs, the periodic lattice is now fluctuating. Importantly, the motion of particles purely as a result of the barostat action depends on their distance from the central simulation box and is thus unbounded (see Figure 1). By contrast, in the toroidal view particles stay in the box with effectively bounded displacements caused by barostat position rescaling. These differences between the toroidal and lattice views seem to have caused some confusion, as there are at least three different algorithms currently in use to unwrap trajectories of constant-pressure MD simulations.

Here, we review and compare the different schemes proposed for trajectory unwrapping at constant pressure (section 2). We use analytic calculations and numerical examples to demonstrate that lattice-preserving unwrapping schemes give rise to unwrapped trajectories with exaggerated fluctuations when used to unwrap NPT simulation data. In extreme cases, the dynamics of these unwrapped trajectories differs sharply from the dynamics of the associated wrapped trajectories (sections 3 and 5). As a consequence, diffusion coefficient estimates are compromised, an effect that becomes apparent already for bulk water at ambient conditions simulated in the NPT ensemble over a microsecond time scale. By contrast, we find that a recently proposed off-lattice unwrapping scheme^2^ preserves the statistical properties of the wrapped trajectory and should therefore be preferred for the calculation of translational diffusion coefficients. However, because the scheme does not adhere to the lattice view, it does not preserve distances.^3^ Molecules should thus first be made “whole” and then unwrapped, e.g., according to their center of mass. We conclude by giving guidance to practitioners on how to extract reliable diffusion coefficient estimates from constant-pressure MD simulations (sections 5.5 and 6).

## 2 Unwrapping Algorithms

### 2.1 Heuristic Lattice-View (HLAT) Scheme

Some MD simulation and visualization software packages implement a lattice-preserving unwrapping scheme (see, e.g., trjconv in GROMACS^4^ and cpptraj in Ambertools^5^), which in one dimension (1D) can be cast into the following form:
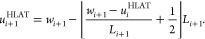
1Here, *w*_*i*_ denotes the wrapped position of a particle inside the simulation box of width *L*_*i*_ at integration step *i* corresponding to time *t*_*i*_, *u*_*i*_^HLAT^ is the corresponding unwrapped position predicted by the HLAT scheme (called the “heuristic scheme” in refs (2) and (3)), and  denotes the floor function. This scheme defines the unwrapped position at time *i* + 1 as the particular lattice image of the wrapped position that minimizes the unwrapped displacement from time *i* to *i* + 1, making it intuitively appealing. In ref (2), however, it was shown that in simulations at constant pressure the above scheme occasionally unwraps particles into the wrong box, which results in an artificial speed up of the particles. This observation was later confirmed in ref (3).

### 2.2 Toroidal-View-Preserving (TOR) Scheme

After exposing the shortcomings of the HLAT scheme, three of the authors of the present paper proposed an alternative unwrapping scheme, which resolves the issues of eq 1 and translates to the following evolution equation in 1D:^2^
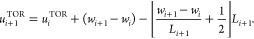
2Taking a toroidal view of PBCs, the TOR scheme considers minimal displacement vectors within the simulation box, which are added together to form an unwrapped trajectory. By design, it therefore preserves the dynamics of the wrapped trajectory. However, the TOR scheme should only be used to unwrap the trajectories of single particles, such as the center of mass of a molecule or a well-chosen reference atom. If the scheme is applied separately to multiple atoms of the same molecule, whose intramolecular bonds cross the periodic boundaries, then the atoms in question get incorrectly displaced with respect to each other, resulting in an unphysical stretching of the bonds connecting them together.^3^ Therefore, molecules should first be made “whole” and then unwrapped.

We note that the TOR scheme (eq 2) appeals to the theory of diffusion coefficients *D* in terms of the autocorrelation function of the velocity *v*(*t*), which in one dimension leads to the Green–Kubo relation^6^*D* = lim_ϵ→0^+^_∫_0_^∞^d*t* exp(−*ϵt*)⟨*v*(*t*)*v*(0)⟩. From this expression, one obtains the Einstein relation *D* = lim_*t*→∞_⟨[*u*(*t*)–*u*(0)]^2^⟩/(2*t*) by writing the position *u*(*t*) as a sum of infinitesimal displacements, *u*(*t*) = *u*(0) + ∫_0_^*t*^d*t*′*v*(*t*′). In analogy, the TOR scheme (eq 2) adds up the minimal displacements between saved configurations to construct an unwrapped trajectory *u*(*t*).

### 2.3 Modern Lattice-View (LAT) Scheme

An alternative to the HLAT scheme, which takes a lattice view of PBCs without succumbing to the known shortcomings of HLAT, is implemented in the qwrap^7^ software package. To our knowledge, this scheme was never explicitly documented in the literature prior to implementation, but the LAMMPS simulation software^8^ uses it to write out unwrapped coordinates.

In the lattice view, crossing the periodic boundaries corresponds to shifting the identity of the particle in the central box to one of its lattice images. The LAT unwrapping scheme keeps track of these shifts using integer image numbers *n*_*i*_ that indicate how many periodic images the current wrapped coordinates are away from the original, unwrapped particle. The image number *n*_*i*_ can be obtained either by explicit bookkeeping of image changes due to wrapping (as done by the remap function of LAMMPS), or by detecting large jumps in the wrapped coordinates (as done by the qunwrap feature of qwrap). In both cases, the unwrapped coordinate can be obtained as a lattice image of its wrapped counterpart, i.e.,
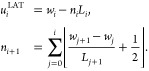
3This is done in qwrap, and in LAMMPS whenever unwrapped coordinates are necessary, such as for output or for use by the Colvars library.^9^

Recently, Kulke and Vermaas^3^ proposed a correction to the TOR scheme with the aim to preserve the underlying lattice structure. Their scheme takes the following form in 1D:
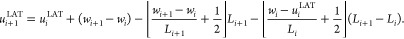
4However, in hindsight it turns out that eq 4 is equivalent to the earlier LAT scheme (eq 3). This can be seen by substituting eq 3 into eq 4, giving 
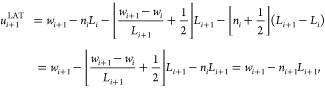
where we exploited the relation  with , an integer number in the second step. For this reason, we make no distinction between eqs 3 and 4 and refer to them both as the LAT scheme.

In what follows, we restrict our discussion to the comparison of the TOR and LAT schemes, as the HLAT scheme has already been established as faulty.

## 3 Theory

Here, we describe a minimal stochastic model of a diffusive particle inside a fluctuating box with PBCs, which we use to generate numerical data and to highlight the differences between the unwrapping schemes via analytic calculations. We also develop and identify appropriate (re)wrapping schemes for the TOR and LAT schemes, respectively. Finally, we derive an upper bound for the frequency with which particle coordinates should be sampled to make sure that all boundary crossings are accounted for.

### 3.1 Minimal Stochastic Model

The 1D Gaussian model was introduced in ref (2) and provides a minimal theoretical description of constant-pressure MD simulations. It consists of a Wiener process *w* that evolves between two periodic boundaries, located at ± *L*_*i*_/2 at time integration step *i*, which are themselves modeled as Gaussian white noise. Due to box length fluctuations, the value of the process gets rescaled in each time step, after which a diffusive displacement is performed. The model gives rise to the following wrapped trajectory:
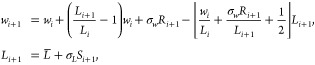
5where  denote uncorrelated normally distributed random variables with zero mean and unit variance, *L* is the average length of the 1D simulation box, and σ_*w*_ and σ_*L*_ determine the noise amplitudes of the random processes driving particle diffusion and box fluctuations, respectively.

In the absence of wrapping events, the displacements resulting from box rescaling and diffusion would give rise to a trajectory of the following form:
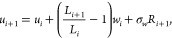
6which can be regarded as the unwrapped partner trajectory to *w* of eq 5. Note that the second term on the right-hand side of eq 6 represents multiplicative noise, as can best be seen in the limit σ_*L*_ ≪ *L*, where we have  with . Notably, this multiplicative-noise term is also present in eq 5, which makes both *w* and *u* distinctively different from an ordinary Wiener process. Yet, because the noise amplitude is only proportional to *w*_*i*_ (and not *u*_*i*_), it remains bounded and does not overshadow the diffusive process.

### 3.2 Differences Between Unwrapping Schemes

Unwrapping the wrapped trajectory of eq 5 using the TOR scheme results in an unwrapped trajectory that coincides with eq 6. This can be demonstrated by substituting eq 5 with  into eq 2, giving 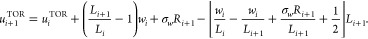
The remaining floor function evaluates to zero as long as σ_*w*_*R*_*i*+1_ ≪ *L*_*i*+1_ and |*L*_*i*+1_ – *L*_*i*_| < *L*_*i*+1_/2, which are reasonable assumptions to make for MD simulations when the sampling interval is sufficiently small. We therefore obtain 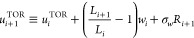
in all practical cases.

By contrast, the LAT scheme evaluates to
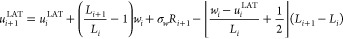
7when applied to the process of eq 5. Here, the last term can be further simplified via eq 3, giving
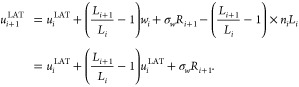
8Comparing eqs 6 and 8, we find that the LAT scheme gives rise to a multiplicative noise term (*L*_*i*+1_/*L*_*i*_ – 1) *u*_*i*_^LAT^ that scales with the unwrapped coordinate. Its magnitude therefore grows without bounds as the particle diffuses away from the origin. The unbounded multiplicative noise in the LAT scheme causes pathological particle dynamics, which becomes apparent when the LAT scheme is used to unwrap trajectories from Brownian dynamics (BD) and MD simulations, as demonstrated below in section 5.

### 3.3 Consistent (Re)Wrapping Schemes

Besides criticizing the undesired effect of intramolecular bond stretching, Kulke and Vermaas^3^ further claimed that the TOR scheme cannot be reversible, because a subsequent wrapping of *u*^TOR^ using “conventional wrapping schemes” does not reproduce the wrapped trajectory *w*. While the authors did not explicitly specify which wrapping schemes they were referring to, we expect a lattice-view scheme, which in 1D reads
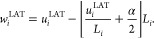
9Here, the value of α depends on the definition of the central unit cell. If it is defined by the interval [0, *L*_*i*_] (as is the case, e.g., in GROMACS^4^) then α = 0, whereas for cells fluctuating symmetrically around the origin, i.e., [−*L*_*i*_/2, *L*_*i*_/2], one has α = 1 (this is the convention that LAMMPS^8^ and NAMD^10^ adhere to). The scheme in eq 9 assumes that at each time integration step *i* the wrapped and unwrapped trajectories are identical up to an integer number of box lengths *L*_*i*_, consistent with the lattice view of eq 3. Equation 9 should therefore be able to perfectly rewrap a trajectory generated by the LAT unwrapping scheme. In fact, substituting eq 7 into eq 9 with *n* = (*w*_*i*_^LAT^ – *u*_*i*_^LAT^)/*L*_*i*_ + 1/2 and α = 1 gives 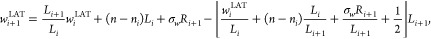
which coincides with eq 5 because 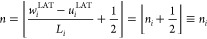
must hold. In light of the fact that the TOR and LAT unwrapping schemes give different results, it is apparent that eq 9 cannot be used to correctly rewrap *u*^TOR^.

To construct a (re)wrapping scheme consistent with the TOR unwrapping scheme, we backtrace the displacements *u*_*i*+1_^TOR^ – *u*_*i*_^TOR^ to reconstruct the wrapped trajectory in an iterative manner as follows:
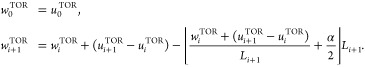
10Whenever the trajectory *w*_*i*_^TOR^ + (*u*_*i*+1_^TOR^ – *u*_*i*_^TOR^) crosses the periodic boundaries, it gets shifted back into the central box with the help of the last term. Substituting eq 6 into eq 10 gives rise to eq 5, as expected. Equations 9 and 10, and their relations to the LAT and TOR unwrapping schemes, are verified with the help of numerical data in section 5.

### 3.4 Upper Bound for the Time Interval Between Sampled Structures in MD Simulations

In section 3.2 we made the assumption that σ_*w*_*R*_*i*+1_ ≪ *L*_*i*+1_ must hold, and argued for its validity for MD simulations at sufficiently small sampling intervals Δ*t*. Very roughly, for a particle with mass *m* and diffusion coefficient *D* we require *D*Δ*t* ≪ *L*^2^ and Δ*t*^2^ ≪ β*m**L*^2^ in the regimes dominated by diffusion and inertia, respectively. Here, β^–1^ = *k*_B_*T* denotes the thermal energy scale, *k*_B_ is the Boltzmann constant and *T* is the absolute temperature. To obtain a quantitative estimate of an upper bound for Δ*t*, we consider the probability *P* for one or more of the *N* particles in the simulation box to travel a distance greater than *L*/2 within Δ*t* along any of the three Cartesian coordinates, resulting in an incorrect unwrapping event in at least one of the *t*_total_/Δ*t* sampled frames, i.e.,
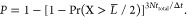
11Here, *t*_total_ is the total simulation time and Pr(*X* > *L*/2) denotes the probability for a single particle in 1D to move either ballistically or diffusively in the time interval Δ*t* by more than *L*/2 in either direction. We can estimate this probability as follows: 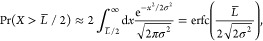
where erfc(·) denotes the complementary error function and 
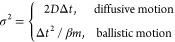
depends on the dominating particle dynamics. For sufficiently small sampling intervals Δ*t*, the probabilities *P* and Pr(*X* > *L*/2) are also small, which allows us to reduce eq 11 to *P* ≈ 3 *N*Pr(*X* > *L*/2) *t*_total_/Δ*t* and replace erfc(·) with its asymptotic expansion for large arguments. Setting *P* = *ε* ≪ 1 results in the expression
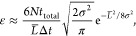
12which can be used to estimate how large the sampling interval Δ*t* can be chosen for a specific MD simulation setup without skewing the resulting unwrapped trajectory due to particles crossing the periodic boundaries.

Solving eq 12 for Δ*t* in the ballistic (σ^2^ = Δ*t*^2^/β*m*) and diffusive limit (σ^2^ = 2*D*Δ*t*) gives the requirements
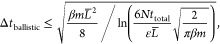
13
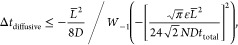
14respectively, where *W*_–1_(·) denotes the lower branch of the Lambert *W* function, which can be expanded for small arguments to give *W*_–1_(*z* → 0^–^) ∼ – ln(−1/*z*) – ln(ln(−1/*z*)) – ln (ln(−1/*z*))/ln(−1/*z*).^11^ For *ε* = 10^–2^ and *N* ≈ 33.3 nm^–3^*L*^3^ TIP3P water molecules with *D* ≈ 6 nm^2^ ns^–1^^12^ and *m* ≈ 18 g mol^–1^ at *T* = 300 K, eqs 13 and 14 evaluate to Δ*t*_ballistic_ ≈ 0.48 ps, 0.94 ps, 1.4 ps and Δ*t*_diffusive_ ≈ 2.9 ps, 11 ps, 25 ps for cubic simulation boxes with edge lengths *L* = 2.5 nm, 5 nm, and 7.5 nm, respectively. For water at ambient conditions,^2^ one can safely use the Δ*t*_diffusive_ bound for times Δ*t* ≥ 1 ps.

## 4 Methods

### 4.1 MD Simulation of TIP4P-D Water with GROMACS

We made use of a 1 μs constant-pressure simulation of 515 TIP4P-D water molecules^13^ in a cubic box with an average edge length of *L* ≈ 2.5 nm, which was previously reported on in ref (2). The simulation was run using GROMACS 2018.6^4^ with a 2 fs integration time step, and particle-mesh Ewald electrostatics^14^ with a 1.2 nm real-space cutoff. The SETTLE algorithm was used to keep water molecules rigid.^15^ The production run commenced after a 100 ps initial equilibration at constant volume and a subsequent 5 ns equilibration run at constant pressure. Temperature and pressure were maintained at 300 K and 1 bar throughout the entire simulation using the velocity-rescaling thermostat^16^ (τ_*T*_ = 1 ps) and the Parrinello–Rahman barostat^17^ (τ_*p*_ = 5 ps), respectively. Particle coordinates were sampled every Δ*t* = 1 ps. For the sake of comparison, we also considered a 1 μs constant-volume simulation of the system described above, which was equilibrated and run in an identical manner.^2^

### 4.2 MD Simulation of SPC/E Water with LAMMPS

We generated a set of wrapped and unwrapped trajectories of 511 SPC/E water molecules^18^ at ambient conditions using the LAMMPS package stable release from September 29, 2021 (update 3).^8,19^ The simulation was performed at constant pressure in a cubic box with an average edge length of *L* ≈ 2.5 nm. The SHAKE algorithm^20^ was used to constrain the intramolecular bonds and angles at an accuracy tolerance of 10^–4^. The particle-particle particle-mesh solver^21^ with a relative force error accuracy of 10^–4^ was used to compute long-range Coulombic interactions, where the cutoff distance in real space was set to 9.8 Å. Equilibration consisted of a 15 ns run in the NVT ensemble, followed by a 20 ns run in the NPT ensemble. Temperature and pressure were maintained at 300 K and 1 bar using the Nosé–Hoover thermostat and barostat^22,23^ with damping coefficients of 100 and 1000 fs, respectively. The 1 μs production run in the NPT ensemble was performed using the same thermostat and barostat coefficients, and a 1 fs integration time step. Particle coordinates of the wrapped and unwrapped trajectory were sampled every Δ*t* = 1 ps via the dump command.

### 4.3 MD Simulation of TIP3P Water with NAMD

We generated an unwrapped trajectory of 826 TIP3P water molecules^24^ at ambient conditions in a cubic periodic box with *L* ≈ 2.9 nm, using NAMD version 3.^10^ A time step of 2 fs was used. Temperature was maintained at 300 K using underdamped Langevin dynamics with a damping time of 1 ps. Pressure was set to 1 bar using the Nosé–Hoover Langevin piston method as implemented in NAMD,^25^ with a piston period of 200 fs and a decay time of 100 fs. Water molecules were kept rigid using the SETTLE algorithm.^15^ Long-range electrostatic interactions were computed using the Particle-Mesh Ewald method, with a 12 Å cutoff for the real-space part. The same cutoff was applied to Lennard-Jones potentials, with force-switching for a continuous decay of the force to zero. Particle coordinates were sampled every Δ*t* = 1 ps for 900 ns in total, this slightly shorter duration being the result of numerical instability (see further section 5.3). To obtain a corresponding wrapped trajectory, we chose to wrap the NAMD output trajectory using eq 9.

## 5 Results and Discussion

### 5.1 Brownian Dynamics Simulations

To verify the analytic predictions of section 3, we evaluated eqs 5, 6, and 8 in an iterative fashion to generate a wrapped trajectory *w*, and two unwrapped trajectories *u* and u*. The wrapped trajectory was unwrapped using the TOR and LAT schemes (eqs 2 and 4, respectively), and the resulting unwrapped trajectories were compared to the corresponding *u* and u* realizations. We used random initial positions *w*_0_ = *u*_0_ = *u*_0_^*^ = *L*(*R*′ – 1/2) with  uniformly distributed on the interval [0,1], and fixed parameter values of σ_*L*_ = 0.1*L* and σ_*w*_ = 0.05*L* .

**Figure 2:**
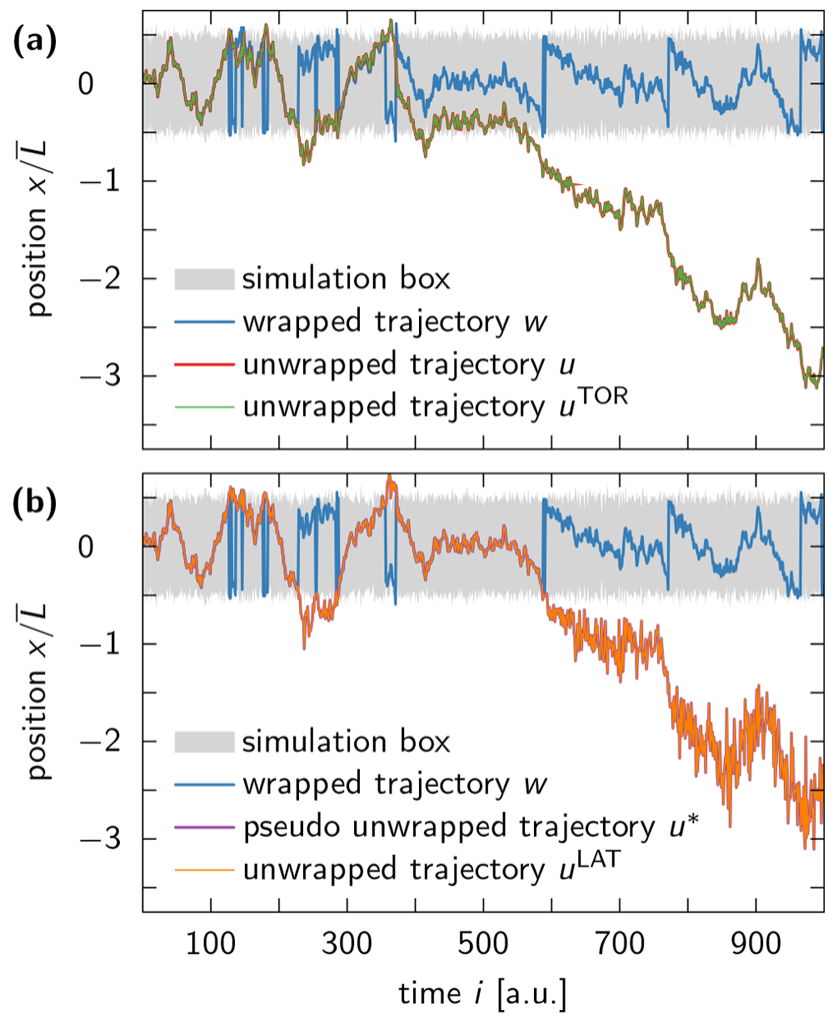
Comparison of the TOR and LAT unwrapping schemes in 1D. (a) TOR unwrapping of trajectory for 1D Gaussian model. While the wrapped trajectory *w* (blue line, eq 5) is confined to the simulation box (gray shaded area), its unwrapped partner trajectory *u* (red line, eq 6) can traverse arbitrarily far from their common initial position. The TOR unwrapping scheme (eq 2), when applied to *w*, produces a trajectory *u*^TOR^ (green line), which completely overlaps with *u*. Note that the unwrapped trajectories *u* and *u*^TOR^ are not “on lattice” in NPT simulations. As a result, they may not coincide with the wrapped trajectory *w* in revisits to the central simulation box, as seen around time 500. (b) LAT unwrapping of the same trajectory as in (a). The unwrapped trajectory *u*^LAT^ (orange line) generated by the LAT unwrapping scheme (eq 4) coincides with the pseudo unwrapped trajectory u* (purple line, eq 8) and exhibits the same exaggerated fluctuations away from the central box.

Figure 2 displays a representative set of trajectories that were generated as described above. In accordance with our predictions in section 3.2, the unwrapped trajectory *u*^LAT^ associated with the LAT scheme exhibits the same position-dependent fluctuations that can be found in u* (eq 8), which increase with the distance to the origin in stark contrast to the dynamics of the wrapped trajectory. Meanwhile, the unwrapped trajectory *u*^TOR^ generated by the TOR scheme shows moderate fluctuations and completely overlaps with *u*, as expected. A visual comparison of trajectory segments between two boundary-crossing events demonstrates that *u*^TOR^ perfectly captures the trends observed in *w*. The same cannot be said about *u*^LAT^.

**Figure 3:**
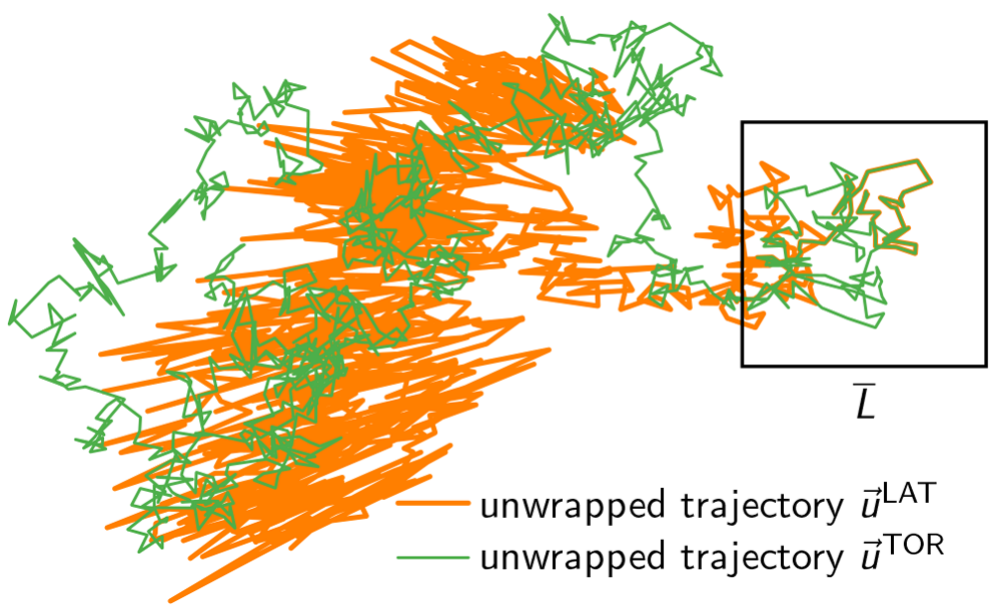
Comparison of the TOR and LAT unwrapping schemes in 2D. A wrapped trajectory *w⃗* of the Gaussian model (not shown) was unwrapped using the TOR and LAT schemes, which resulted in the unwrapped trajectories *u⃗*^TOR^ (green line) and *u⃗*^LAT^ (orange line), respectively. While *u⃗*^TOR^ is visually indistinguishable from an ordinary diffusive trajectory anywhere in the plane, *u⃗*^LAT^ is strongly affected by box fluctuations after leaving the central simulation box (average size shown as black square). Note that the apparent noise in *u⃗*^LAT^ grows with the distance from the central box and becomes anisotropic, with position fluctuations emanating in a star-like fashion from the origin.

The nondiffusivity of *u*^LAT^ is even more pronounced in higher dimensions, as illustrated in Figure 3, where we combine two 1D trajectories of the Gaussian model, *w*_*x*_ and *w*_*y*_, to construct a two-dimensional (2D) wrapped trajectory *w⃗* = (*w*_*x*_, *w*_*y*_)^*T*^. The unwrapped trajectories *u⃗*^TOR^ and *u⃗*^LAT^ were generated by unwrapping each component of *w⃗* separately using the TOR and LAT schemes, respectively. As shown, the apparent noise in the *u⃗*^LAT^ trajectory not only grows with distance from the central simulation box but also becomes anisotropic. Supporting Movie S1 visualizes the evolution of the trajectories and the fluctuations of the simulation box.

**Figure 4:**
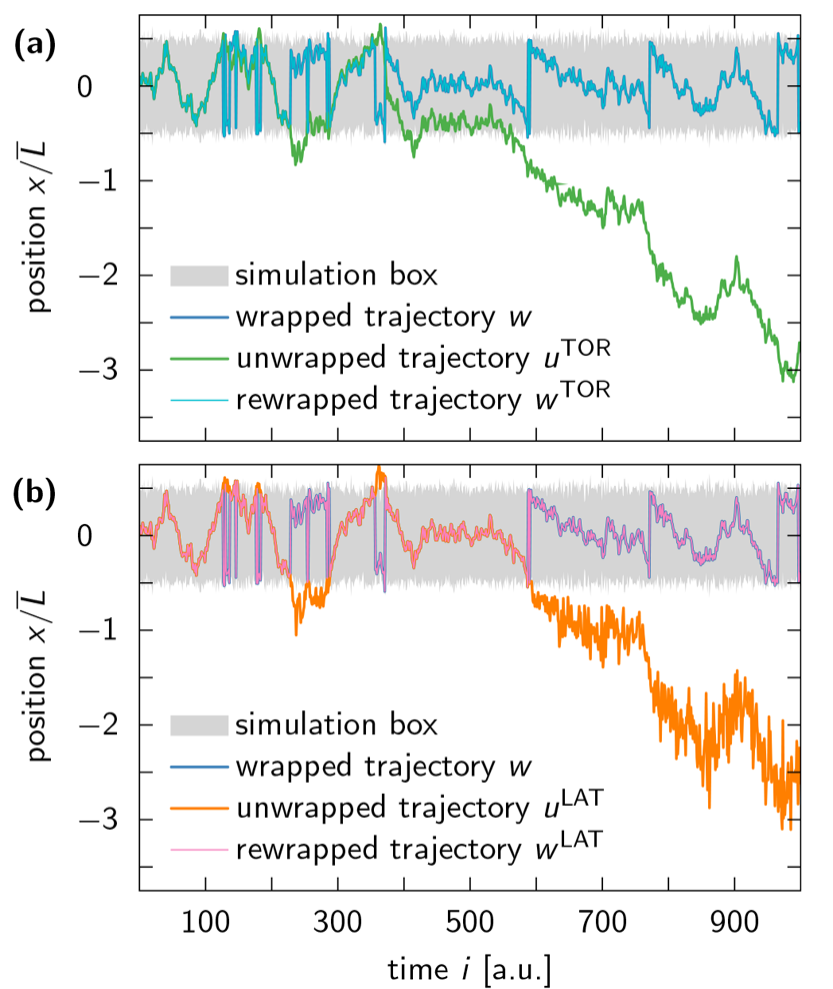
Rewrapping trajectories using appropriate wrapping schemes. (a) The unwrapped trajectory *u*^TOR^ (green line), generated by the TOR scheme, can be perfectly rewrapped inside the simulation box (gray shaded area) using eq 10 with α = 1, as seen by the complete overlap of *w*^TOR^ (cyan line) with the original wrapped trajectory *w* (blue line). (b) Similar results can be achieved for *u*^LAT^ (orange line), generated by the LAT scheme, if it is rewrapped using eq 9 with α = 1. This gives rise to the trajectory *w*^LAT^ (pink line).

Finally, we assessed the ability of eqs 9 and 10 to reverse the operations of the LAT and TOR unwrapping schemes, respectively. In Figure 4, we demonstrate that eq 10 faithfully reproduces the wrapped 1D trajectory *w* when applied to *u*^TOR^. Similarly, we find that eq 9 perfectly rewraps *u*^LAT^ back into the simulation box. It is therefore unsurprising that Kulke and Vermaas^3^ only found their own unwrapping scheme to be reversible with respect to “conventional wrapping schemes” (such as eq 9): the unwrapped trajectories *u*^TOR^ and *u*^LAT^ are different and therefore require different wrapping schemes to be correctly rewrapped into the simulation box.

### 5.2 GROMACS Simulations

In our BD simulations, we could freely choose the amplitude σ_*L*_ of the box fluctuations to highlight the difference between the two unwrapping schemes. In MD simulations, however, box fluctuations for aqueous systems at ambient conditions are generally well below one percent of the average edge length, so the amplified fluctuations in *u*^LAT^ are much more subtle. To test whether we can identify considerable differences between the TOR and LAT schemes in MD simulations, we analyzed the wrapped trajectories of TIP4P-D water in a small, fluctuating, cubic box, as reported previously in ref (2) (see section 4.1 for technical details).

**Figure 5:**
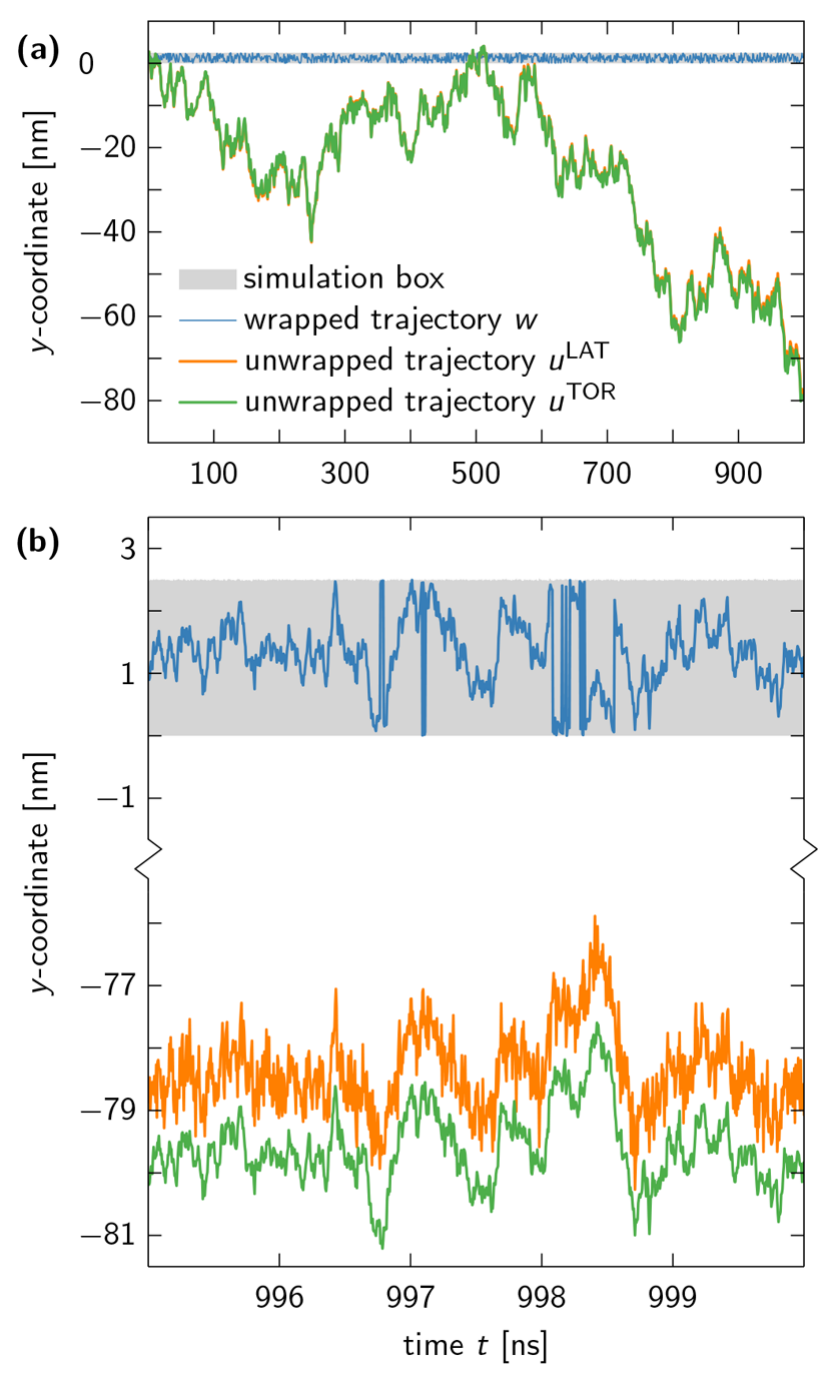
Trajectory of an oxygen atom of a TIP4P-D water molecule along a single coordinate axis. (a) The wrapped trajectory *w* (blue line) is unwrapped via the TOR and LAT schemes, resulting in *u*^TOR^ (green line) and *u*^LAT^ (orange line), respectively. The two unwrapped trajectories seem almost identical, because box fluctuations in MD simulations of water at ambient conditions are small compared to the dimensions of the simulation box (gray shaded area). (b) However, a zoom-in on the last 5 ns of the trajectory reveals that *u*^LAT^ exhibits larger fluctuations between subsequent time frames than *u*^TOR^ and *w*. The enhanced noise in *u*^LAT^ is indicative of the unbounded multiplicative noise associated with the LAT unwrapping scheme.

In Figure 5, we plot the *y*-component of the trajectory of an oxygen atom in a water molecule that managed to diffuse more than 30 box edge lengths away from the central simulation box. At first glance, the unwrapped trajectories produced by the TOR and LAT schemes may seem identical, but when we zoom in on the last few nanoseconds of the trajectory, we find *u*^LAT^ to have the same exaggerated fluctuations as observed in our BD simulations. By contrast, *u*^TOR^ visually reproduces the features of the wrapped trajectory *w*.

**Figure 6:**
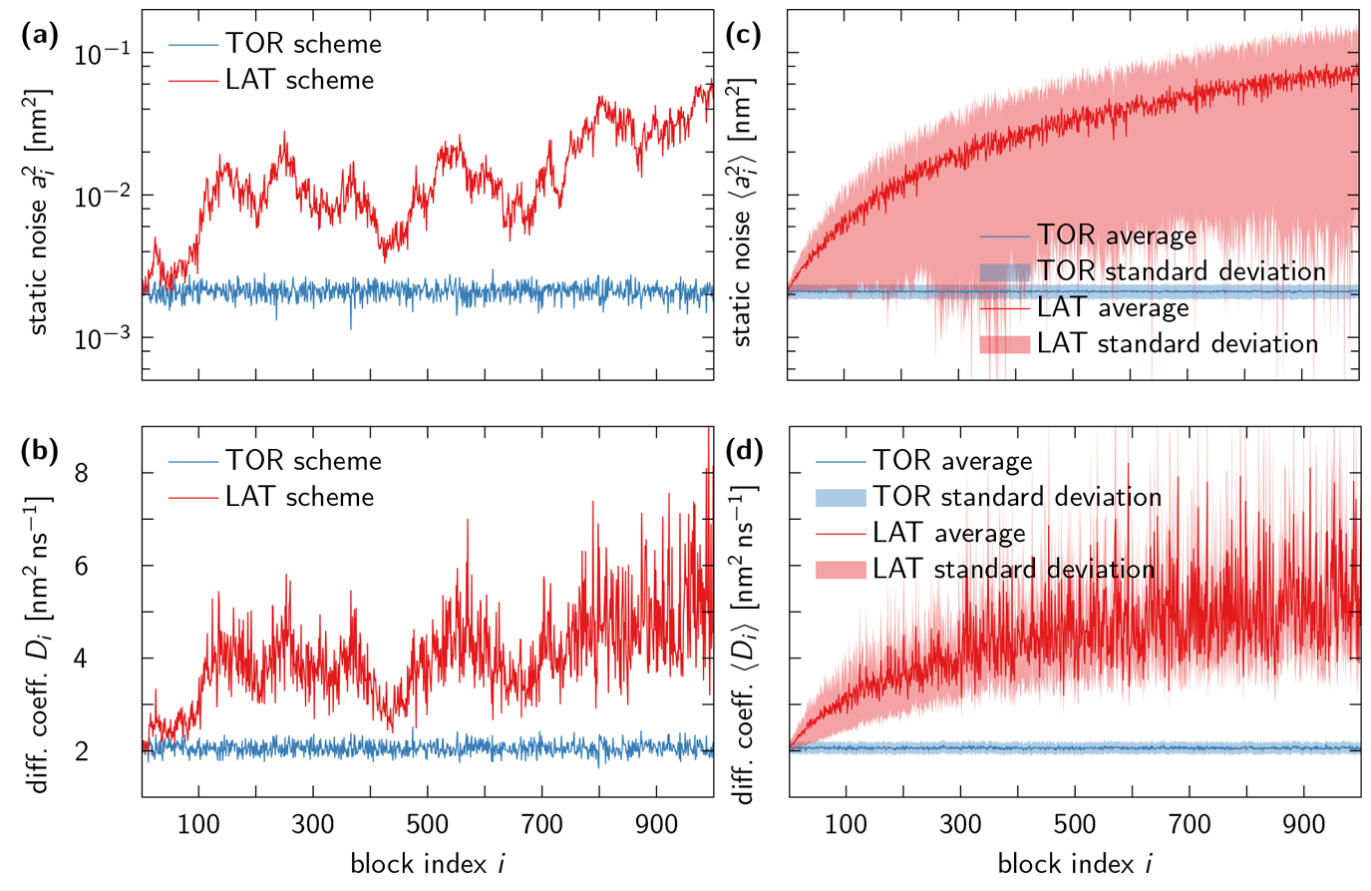
Diffusion coefficient estimates are robust for TOR unwrapping but compromised by LAT unwrapping. Shown are results for the static noise and diffusion coefficient estimates obtained using the MLE from trajectories of oxygen atoms in TIP4P-D water saved at a time interval of Δ*t* = 1 ps and divided into 1000 blocks *i* of 1 ns each. (a) Static noise amplitude *a*_*i*_ and (b) diffusion coefficient *D*_*i*_ estimated for each block *i* of the unwrapped trajectory of a single water molecule using the TOR (blue lines) and LAT schemes (red lines). (c) Average static noise amplitude *a*_*i*_ and (d) diffusion coefficient *D*_*i*_ estimates over all water molecules using the TOR (blue) and LAT schemes (red). In (c, d), averages are shown as solid lines and standard deviations as shaded areas.

To quantify the effect that box fluctuations have on unwrapped trajectories, we analyzed the diffusive behavior observed in different trajectory segments. We made use of a maximum likelihood estimator^26^ (MLE) for the diffusion coefficient *D*, which accounts for the fact that a *d*-dimensional diffusive process *X⃗*(*t*) can be corrupted by static noise and dynamic motion blur, resulting in the following mean squared displacement (MSD):
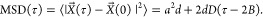
15Here, τ denotes the lag time. For MD simulations, the motion blur coefficient *B* is zero and the vertical intercept *a*^2^ accounts for nondiffusive dynamics at short times.^27^ Note that the MLE does not rely explicitly on estimates for the MSD, but instead exploits the statistics of the increments *X⃗*(*t*_*i*+1_) – *X⃗*(*t*_*i*_). We segmented our unwrapped oxygen trajectories for TIP4P-D water into 1 ns blocks and extracted for each block (with index *i*) estimates for the static noise *a*_*i*_^2^ and the diffusion coefficient *D*_*i*_. Figure 6 presents our results for the TOR and LAT schemes. Unsurprisingly, the TOR scheme gives consistent parameter estimates for all block indices *i*, whereas the estimates for the LAT scheme vary strongly with *i* and tend toward larger values at later times in the trajectory. This behavior is to be expected as the diffusive spread of the water molecules moves them further away from the central simulation box, where artifacts become more pronounced in trajectories associated with the LAT unwrapping scheme.

It should be noted that our results imply a significant difference between global diffusion coefficient estimates obtained for the TOR scheme and the LAT scheme with a Δ*t* = 1 ps time step. Consistent with the analysis of Figure 6, we find the global mean estimates *D*_TOR_ = 2.0602(2) nm^2^ ns^–1^ and *D*_LAT_ = 4.64(3) nm^2^ ns^–1^ by applying the MLE to the full 1 μs trajectory of each oxygen atom and then average over all water molecules, without correcting for finite-size effects.^12,28^ By comparison, we obtained the global mean estimate *D* = 2.0517(2) nm^2^ ns^–1^ for the corresponding NVT simulation (see section 4.1 for technical details). The standard errors of *D*_TOR_, *D*_LAT_, and *D* (in parentheses) were estimated by assuming that the diffusion processes of individual water molecules were uncorrelated. Importantly, we expect the discrepancy between *D*_TOR_ and *D*_LAT_ to grow if the MD simulations are extended beyond the 1 μs used here, because *D* estimates in Figure 6 obtained with the MLE for the LAT unwrapped trajectories slowly increase to ever larger values as the traced particles move further away from the central box (see Figure 6).

### 5.3 LAMMPS and NAMD Simulations

The GROMACS simulation software package exclusively generates wrapped trajectories. These trajectories are typically unwrapped in a postprocessing step using built-in tools like trjconv or third-party software, such as the PBCTools^29^ and qwrap^7^ plugins for VMD.^30^ Other MD simulation codes write out unwrapped trajectories directly, either by default or via user-specified settings, but this raises the question which unwrapping scheme these trajectories correspond to. We therefore analyzed simulation trajectories for SPC/E and TIP3P water generated via the software packages LAMMPS and NAMD, respectively (see sections 4.2 and 4.3 for technical details). NAMD does not, in general, wrap the particle coordinates throughout the simulation, except when writing coordinates to disk, and then only when instructed to do so through the user options wrapAll or wrapWater. LAMMPS, by contrast, allows the user to specify whether the wrapped coordinates, unwrapped coordinates, or both should be written out.

**Figure 7:**
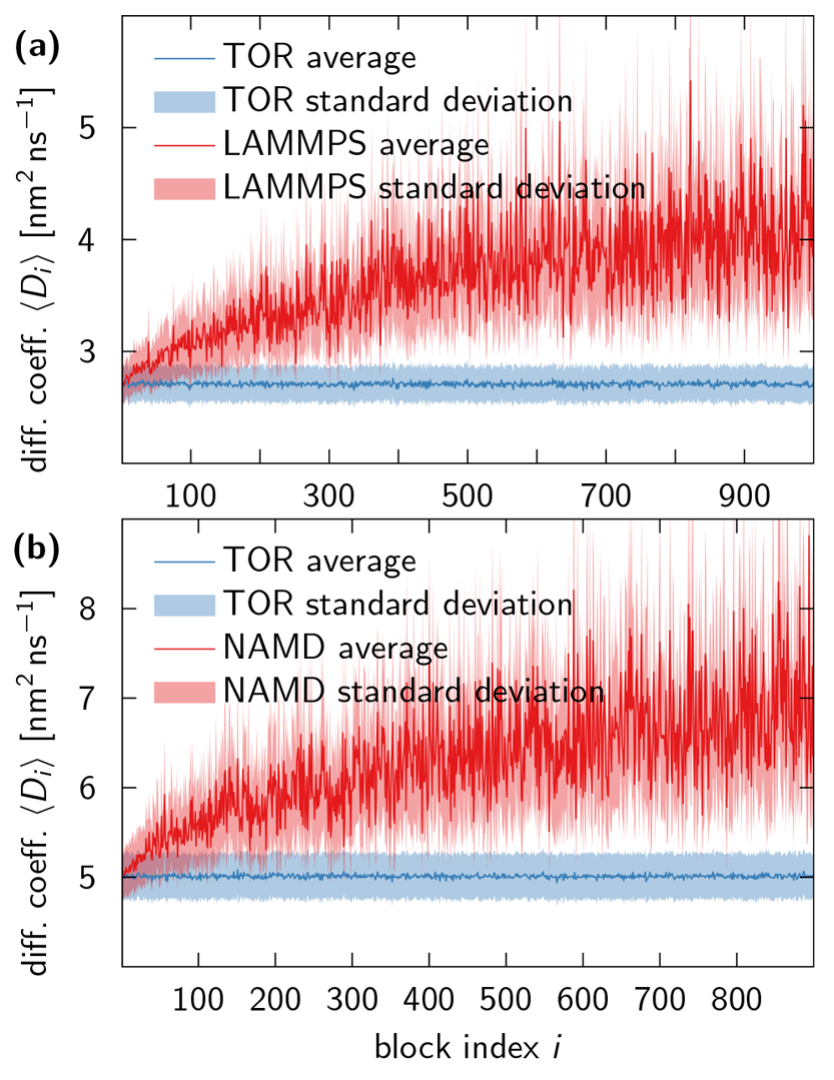
Diffusion coefficient estimates for default-unwrapped NAMD trajectories and automatically unwrapped LAMMPS trajectories are compromised. (a) LAMMPS simulation of SPC/E water. Diffusion coefficients were estimated separately for 1000 consecutive blocks *i*, each 1 ns long, of a continuous trajectory created by writing out “unwrapped” coordinates (red). A corresponding wrapped trajectory, also written out by LAMMPS, was unwrapped using the TOR scheme, and analyzed analogous to the automatically unwrapped LAMMPS trajectory to produce the TOR estimates of the diffusion coefficient (blue). (b) NAMD simulation of TIP3P water. The data analysis procedure was the same as used for the LAMMPS trajectories, except that the wrapped counterpart of the NAMD trajectory was generated using eq 9. Due to the fact that the NAMD trajectory was 100 ns shorter than the LAMMPS trajectory, it was split into 900 blocks. Averages are shown as solid lines and standard deviations as shaded areas.

We segmented the unwrapped trajectories of oxygen atoms generated by LAMMPS and NAMD into 1 ns blocks and analyzed the diffusive dynamics of every block separately, as detailed in section 5.2. This was also done to the corresponding wrapped partner trajectories, after unwrapping them via the TOR unwrapping scheme. The resulting diffusion coefficient estimates as functions of the time window are shown in Figure 7.

We find that the diffusion coefficients calculated directly from the unwrapped trajectories steadily diverge to ever larger values as a function of time. By contrast, the wrapped trajectories, which were unwrapped using the TOR scheme, produce robust diffusion coefficient estimates. The behavior in Figure 7 mirrors that seen in Figures 6(c) and 6(d), and is fully consistent with LAMMPS and NAMD producing unwrapped NPT trajectories that are “on lattice” and thus not suitable for the estimation of diffusion coefficients. Indeed, the global mean diffusion coefficients obtained by analyzing the entire unwrapped trajectories produced directly (i.e., without division into blocks) by LAMMPS and NAMD are inconsistent with the results for early and late blocks. The global mean is *D*_LAMMPS_ = 3.75(1) nm^2^ ns^–1^ for SPC/E water in the LAMMPS simulation, whereas early and late blocks give values around 2.7 nm^2^ ns^–1^ and 4 nm^2^ ns^–1^, respectively [see Figure 7(a)]. For TIP3P water in the NAMD simulations, the global mean is *D*_NAMD_ = 6.50(2) nm^2^ ns^–1^, with early and late blocks around 5 nm^2^ ns^–1^ and 7 nm^2^ ns^–1^, respectively [see Figure 7(b)]. By contrast, the global mean *D*_TOR_ = 2.7078(2) nm^2^ ns^–1^ obtained after TOR unwrapping of the wrapped LAMMPS trajectory is consistent with the respective block estimates, as is *D*_TOR_ = 5.0132(3) nm^2^ ns^–1^ obtained for the NAMD trajectory. Note that we did not correct for system-size effects on the self-diffusion coefficients.^12,28^

The NAMD simulation became numerically unstable as particle coordinates and their barostat-induced fluctuations became large. While such a small box with less than 1000 water molecules can be seen as an extreme example, this phenomenon highlights a benefit of propagating wrapped coordinates internally during the simulation, which is to make the best use of limited floating-point precision, especially in mixed-precision GPU software. In NAMD this behavior can be approached by enabling the wrapAll or wrapWater options, resulting in all coordinates or specifically water molecules being wrapped at every restart.

### 5.4 Are Published Diffusion Coefficients from NPT Simulations Compromised by Incorrect Unwrapping?

To assess the possible impact of LAT unwrapping on diffusion coefficients obtained from NPT simulations in earlier studies, it is important to recognize that the vast majority of *D* estimates were calculated from ordinary least-squares (OLS) fits of straight lines to the MSD. The fit region is usually chosen “by eye” to cover a window in which the MSD grows more or less linearly with time. Considering the fact that, to a rough approximation, LAT trajectories make ever larger jumps about TOR trajectories, it is conceivable that simple *D* estimates obtained from OLS fits to the MSD are less impacted by LAT unwrapping than the more sophisticated MLE used in the analysis above.

**Figure 8:**
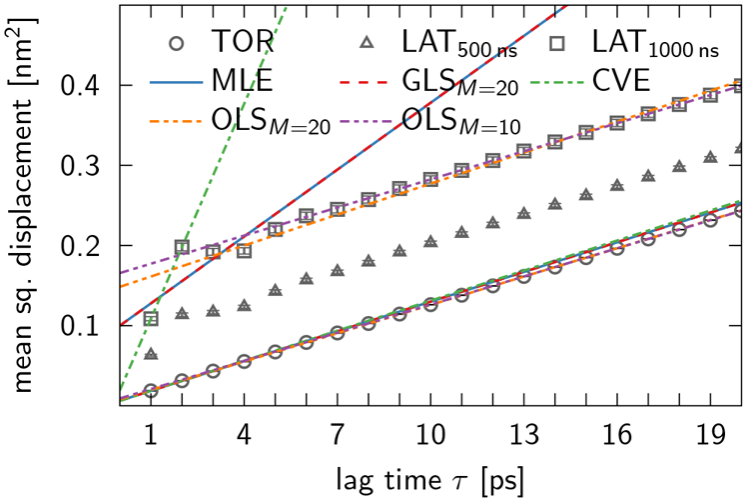
LAT unwrapping compromises the MSD of TIP4P-D water trajectories at short lag times τ. While the MSD of TOR unwrapped trajectories (circles) is linear at all lag times, its counterpart for LAT trajectories (triangles for the first 500 ns of the simulation and squares for the full simulation time) displays oscillations for small τ, which grow in size with the length of the trajectories. The corresponding error bars are smaller than the markers. Lines represent eq 15 with values of *a*^2^ and *D* that were calculated using various estimators (see legend) for a time step of Δ*t* = 1 ps. The associated mean diffusion coefficients *D*_TOR_ and *D*_LAT_ are listed in Table 1.

In Figure 8, we present the MSD of LAT and TOR trajectories (averaged over all trajectories) at different lag times τ = *m*Δ*t* for the TIP4P-D water data analyzed in section 5.2. The single-trajectory MSD values were estimated as 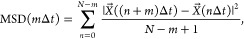
with *N* = 10^6^ data points for a time step of Δ*t* = 1 ps. The MSD obtained from the LAT unwrapped trajectories oscillates at short lag times and then approaches a straight line. Halving the simulation time to 500 ns reduces the magnitude of the oscillations at short times, consistent with the observation that the amplitude of oscillations in the LAT unwrapped trajectories tends to increase with their duration (Figure 3). At long lag times, however, the slope of the MSD from LAT unwrapping approaches that obtained from TOR unwrapping. This is confirmed by OLS fits, where we found good agreement between diffusion coefficient estimates for the TOR and LAT data. These findings explain why no significant difference was found between *D*_TOR_ and *D*_LAT_ in ref (3). For completeness, we also analyzed the unwrapped trajectories using a generalized least-squares estimator^27^ (GLS) and a covariance-based estimator^31^ (CVE). The resulting estimates of the diffusion coefficient of TIP4P-D water (without correcting for system-size effects^12,28^) are listed in Table 1. Note that the slight discrepancy between the MSD for the TOR unwrapped trajectories at long lag times τ and eq 15, evaluated using the estimates of MLE, GLS, and CVE (see Figure 8), results from the fact that the dynamics of TIP4P-D water is not perfectly diffusive at Δ*t* = 1 ps. This was discovered and extensively discussed in ref (27), where a time step of Δ*t* = 10 ps was identified as optimal, in the sense that it is short enough to minimize statistical errors but long enough to ensure diffusive dynamics. In fact, if the LAT and TOR trajectories, which were unwrapped at Δ*t* = 1 ps, are subsampled at Δ*t* = 10 ps and then reanalyzed, one gets fairly similar diffusion coefficients for both unwrapping schemes and all estimators considered here (see Table 1). The TOR-trajectory estimates of MLE, GLS, and CVE all lie within their respective statistical uncertainties.

**Table 1:** Mean Diffusion Coefficients of TIP4P-D Water Calculated Using Various Estimators^a^

			Δ*t* = 1 ps time step		Δ*t* = 10 ps time step	
estimator	*m*_init_	*m*_end_	*D*_TOR_	*D*_LAT_	*D*_TOR_	*D*_LAT_
MLE^26^			2.0602(2)	4.64(3)	1.9523(4)	1.9442(5)
GLS^27^	1	20	2.0602(2)	4.64(3)	1.9523(4)	1.9442(5)
CVE^31^			2.0838(2)	14.9(4)	1.9526(4)	1.9381(7)
OLS_*M*=20_	1	20	1.9646(3)	2.147(5)	1.9475(9)	1.9465(9)
OLS_*M*=10_	11	20	1.9518(4)	1.9487(4)	1.947(1)	1.948(1)
MLE			*D*_NVT_ = 2.0517(2)		*D*_NVT_ = 1.9507(4)	

a The GLS and OLS estimators were evaluated for *M* = *m*_end_ – *m*_init_ + 1 MSD values at lag times *τ* = *m* Δ*t* with *m* ∈ [*m*_init_, *m*_end_]. All diffusion coefficients are uncorrected for finite-size effects and have units of nm^2^ ns^–1^. Uncertainties in the last significant digit are listed in parentheses and correspond to standard errors of the mean.

As a further consistency check, we calculated the TIP4P-D water diffusion coefficient also in the NVT ensemble, where unwrapping is unambiguous and the TOR and LAT schemes are equivalent. The NVT simulations were performed for a system of the same size and temperature, with a volume calibrated to produce a pressure close to that of the NPT simulations.^2^ We found that the diffusion coefficient estimates obtained with the MLE for trajectories in the NVT ensemble sampled at time steps of Δ*t* = 1 and 10 ps are both in very close agreement with the respective estimates in the NPT ensemble obtained with TOR unwrapping (Table 1). This consistency indicates that the box-size fluctuations caused by the barostat did not noticeably impact the statistical properties of the molecular diffusion process for NPT trajectories unwrapped with the TOR scheme.

Reassuringly, the results in Figure 8 and Table 1 suggest that earlier diffusion coefficients may actually be less impacted by LAT unwrapping than the results of Figures 6 and 7 suggest, for the simple reason that in the MD community, straight-line fits to the MSD are still commonly used to estimate *D*. Moreover, early simulations tended to be comparably short, so that particles did not diffuse far from the simulation box at the origin. Going forward, however, we recommend to take advantage of the statistically more efficient MLE^26^ or GLS^27^ estimators applied to TOR-unwrapped trajectories, which preserve the diffusive characteristics also at long times, unlike the LAT trajectories. Combining powerful estimators with TOR-unwrapped trajectories avoids systematic errors and minimizes statistical errors.

### 5.5 Correctly Applying the TOR Scheme to Bonded Atoms

Now that we have established the correctness and consistency of the TOR unwrapping scheme for single particles, we next address possible issues that can arise due to the fact that the scheme does not adhere to the lattice view and therefore cannot preserve distances between particles. This becomes a problem, for example, when the TOR scheme is applied naively to bonded particles.

Kulke and Vermaas^3^ observed that bond lengths only got distorted when the TOR scheme was applied to trajectories generated by the GROMACS software package, whereas NAMD trajectories seemed unaffected. The reason for this discrepancy is the fact that NAMD by default treats molecules as “whole” when writing out data, i.e., the software does not break up molecules that sit on the periodic boundary. A preprocessing step in the analysis of GROMACS data, to make molecules whole prior to unwrapping, will therefore remedy the seeming shortcoming of the TOR scheme observed in ref (3).

Irrespective of the simulation software behind the data to be analyzed, we recommend the following order of operations when unwrapping MD simulation data of molecules:

1. In each frame of the trajectory, make the molecule “whole,” i.e., starting from a chosen reference atom of the molecule, ensure that all covalent bonds correspond to their minimal distance over the periodic images.
2. Calculate the center of mass of the “whole” molecule and, in case the resulting coordinate is located outside of the simulation box, perform a wrapping operation. This generates a wrapped trajectory of the center-of-mass coordinate of the molecule.
3. Unwrap the trajectory of the center-of-mass coordinate using the TOR unwrapping scheme.
4. If needed, the molecule can be reconstructed along the unwrapped center-of-mass trajectory by using the positions of the atoms relative to the center of mass of the “whole” molecule in each frame.

Note that the calculation of the center-of-mass coordinate in step 2 can be avoided by using instead the position of a specific atom as reference, say the oxygen atom of a water molecule. Also note that the estimation of translational diffusion coefficients only requires the tracking of the center of mass or any chosen reference atom.

### 5.6 Pair Diffusion

Finally, one might speculate whether the nonpreservation of distances in the TOR unwrapping scheme affects other observables that rely on unwrapping, such as pair diffusion coefficients. According to theory, the distance vector *X⃗* – *Y⃗* between two independent diffusion processes, *X⃗*(*t*) and *Y⃗*(*t*), is also diffusive with the following diffusion coefficient:
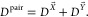
16Here, *D*^*Z⃗*^ denotes the diffusion coefficient of the process *Z⃗*(*t*).

**Figure 9:**
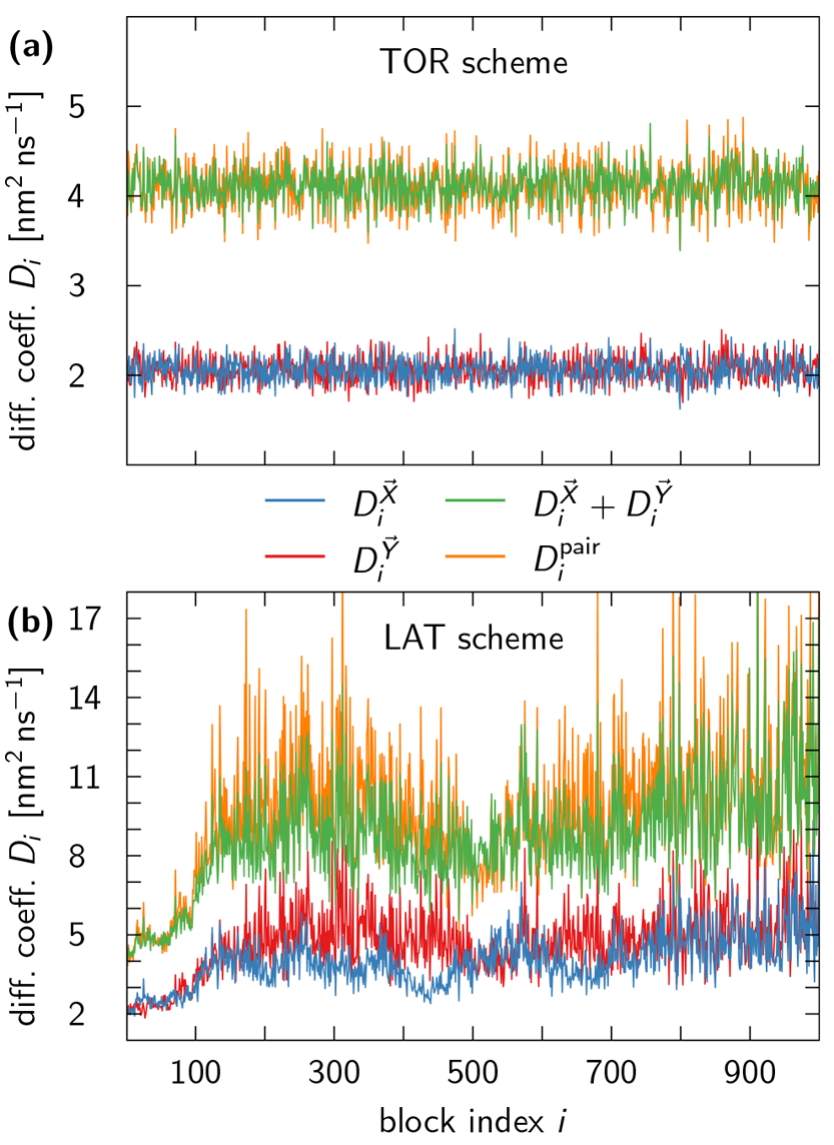
Pair diffusion coefficient estimates for the TOR and LAT scheme both satisfy the additivity relation (eq 16) but are only robust for the TOR scheme. (a) Analogous to Figure 6(a), we analyzed 1 ns segments of two unwrapped trajectories generated by the TOR scheme and extracted the corresponding diffusion coefficients *D*_*i*_ (blue and red lines). The pair diffusion coefficient *D*_*i*_^pair^ (orange line) agrees well with *D*_*i*_^*X⃗*^ + *D*_*i*_^*Y⃗*^ (green line), but numerical discrepancies are due to the nonlinearity of our MLE. (b) Same as in panel (a) for trajectories unwrapped using the LAT scheme.

To test whether pair diffusion is preserved for the TOR scheme, the LAT scheme, or both, we considered two randomly selected TIP4P-D water molecules from the GROMACS MD simulation and analyzed the diffusive behavior of *X⃗*, *Y⃗*, and *X⃗* – *Y⃗*, as described in section 5.2. Figure 9 demonstrates that both unwrapping schemes essentially satisfy eq 16, but only the TOR scheme gives consistent results for all trajectory segments. As for single-particle diffusion, the pair diffusion coefficient obtained by LAT unwrapping tends to grow with time. It is therefore clear that LAT unwrapping and the associated lattice view of the PBCs are not beneficial for the estimate of pair diffusion coefficients in constant-pressure NPT simulations.

A special case of restricted pair diffusion is the end-to-end relaxation of long (bio)polymers. Any motions that extend over the scale of the box will require care in unwrapping of PBCs. For polymer end-to-end relaxations and similar properties, one should first make the molecule “whole” across PBCs using the instantaneous box size and then calculate the relaxation dynamics of, say, the end-to-end distance.

## 6 Conclusions

Unwrapping trajectories of constant-pressure MD simulations is a nontrivial task. One school of thought takes a toroidal view of the PBCs to construct an unwrapped trajectory by adding up minimal displacement vectors at each time step.^2^ Another^3,7^ takes a lattice view of the PBCs and traces the trajectory through the fluctuating lattice of image particles. As a consequence of the fluctuations in box size and shape, and the associated fluctuations in the lattice parameters, the two approaches produce different unwrapped trajectories. We have shown here that the toroidal approach embodied in the TOR algorithm^2^ sacrifices the preservation of interparticle distances to preserve the statistical properties of the wrapped trajectory. In particular, a trajectory created by a diffusion process retains its diffusive character. By contrast, the lattice view taken in the LAT algorithm^3^ preserves distances between particles, but distorts the statistical properties of local dynamics and thus destroys the diffusive character of a wrapped diffusion trajectory.

Our analytic calculations (section 3.2), as well as our results from BD and MD simulations (sections 5.1 and 5.2), demonstrate that the lattice-preserving LAT unwrapping scheme amplifies position-dependent fluctuations that arise in wrapped trajectories of constant-pressure simulations due to barostat position rescaling. Meanwhile, the TOR unwrapping scheme manages to preserve the diffusive character of the wrapped trajectories. These observations are further confirmed by our diffusion analysis of unwrapped MD trajectories, where different segments of TOR trajectories give consistent diffusion coefficient estimates, whereas the diffusive dynamics at the beginning and end of LAT trajectories differ greatly (see Figure 6 for water at ambient conditions).

A surprising conclusion is that the “unwrapped” trajectories written out by MD simulation software like NAMD and LAMMPS for NPT simulations should not be used to calculate diffusion coefficients (section 5.3). The reason is that these trajectories are “on lattice,” i.e., the corresponding wrapped positions are obtained by eq 9. Therefore, as the particles diffuse away from the reference box at the origin, they increasingly pick up the multiplicative noise resulting from box rescaling, as visualized in Figures 2 and 3. To avoid the resulting artifacts (see, e.g., Figure 7) and to obtain accurate diffusion coefficients, the output trajectories should first be wrapped “on lattice” via eq 9 and then unwrapped “off lattice” using the TOR scheme (eq 2).

In light of the fact that particle dynamics in constant-pressure simulations is always affected by a (bounded) barostat-induced multiplicative noise, even in the wrapped trajectory, one might be tempted to construct a postprocessing scheme to remove this noise altogether. However, such sanitation is highly nontrivial and its advantage over performing simulations at constant volume is not evident. For this reason, we instead take a toroidal view of the PBCs and treat the wrapped particle dynamics as the “true” dynamics corresponding to the given simulation ensemble, despite the presence of multiplicative noise. If the multiplicative noise associated with barostat position rescaling is considered an issue, we recommend constant-volume simulations instead of the complex and somewhat arbitrary postprocessing of trajectories generated at constant pressure.

Reassuringly, we expect published studies of diffusion coefficients to be minimally affected by the choice of unwrapping scheme. For one, early simulations tended to be short, making diffusion far beyond the central box and thus unwrapping artifacts rare, in particular for larger molecules. In addition, diffusion coefficients were usually estimated by least-squares fitting to the MSD, which we found to be quite robust at sufficiently long times (section 5.4).

Although current MD simulations of large biological molecules are only minimally affected by the shortcomings of the LAT scheme reported here, we expect our findings to become crucial for the proper analysis of future simulation trajectories on the time scales of milliseconds and beyond. For NPT simulations, we recommend the use of large boxes, for which the time to diffuse over multiple box dimensions is large and the position rescaling effects are small. The latter follows from the decay in the relative fluctuations of the box volume *V* with system size, namely β⟨(*V* – ⟨*V*⟩)^2^ ⟩ = χ_*T*_⟨*V* ⟩, where χ_*T*_ denotes the isothermal compressibility.^32^

For precision calculations of diffusion coefficients and related quantities, one may want to resort to NVT simulations. The choice of an NVT ensemble is advisable in particular for long simulations with small boxes of highly compressible systems with low viscosity, where the relative box-size fluctuations are large and particles can diffuse over many box widths. At constant volume, the lattice and toroidal view of PBCs coincide and unwrapping is unambiguous. If needed, the results from NVT simulations at different volumes can be interpolated to the targeted pressure or rigorously combined into weighted samples of an NPT ensemble.^33,34^ An added advantage of working at constant volume is that the box size and shape entering the large finite-size corrections of translational diffusion coefficients^12,28^ are well defined, whereas for NPT conditions one usually resorts to averages.

## Supporting Information Available

Supporting Movie S1 compares the time evolution of 2D trajectories generated via the TOR and LAT schemes (MP4)
